# Geospatial analysis as a tool to identify target areas for Chagas disease education for healthcare providers

**DOI:** 10.1186/s12879-022-07577-y

**Published:** 2022-07-04

**Authors:** Gerardo J. Pacheco, Lawrence Fulton, Jose Betancourt, Ram Shanmugam, Paula Stigler Granados

**Affiliations:** 1 School of Health Administration, Texas State University, San Marcos, TX 78666 USA; 2 School of Public Health, San Diego State University, San Diego, CA 92182 USA

**Keywords:** Chagas disease, *Trypanosoma cruzi*, American Trypanosomiasis, Neglected tropical diseases, Geospatial analysis, Heart disease

## Abstract

Chagas Disease (CD) is a neglected zoonotic disease of the Americas. It can be fatal if not diagnosed and treated in its early stages. Using geospatial and sensitivity analysis, this study focuses on understanding how to better allocate resources and educational information to areas in the United States, specifically Texas, that have the potential for increased risk of CD cases and the associated costs of addressing the disease. ICD-9 and 10 inpatient hospital diagnostic codes were used to illustrate the salience of potentially missed CD diagnoses (e.g., cardiomyopathic diagnoses) and where these are occurring with more frequency. Coding software along with GIS and Microsoft Excel 3D mapping were used to generate maps to illustrate where there may be a need for increased statewide surveillance and screening of populations at greater risk for CD. The CD cases reported to the Texas Department of State Healthcare Services (TxDSHS) are not homogenously dispersed throughout the state but rather, reveal that the incidences are in clusters and primarily in urban areas, where there is increased access to physician care, CD research and diagnostic capabilities.

## Introduction

Chagas Disease (CD) is a neglected zoonotic disease [1] of the Americas that can be fatal if left undiagnosed and not treated in its early stages. CD accounts for the highest burden of any parasitic disease in the Latin American countries where it is endemic. *Trypanosoma cruzi,* the parasite responsible for Chagas disease*,* is endemic throughout Central and South America and is also found in North America, including Mexico and the Southern United States (U.S.) [2–5]. An estimated 8 million people in Latin America have CD [6]. Over 28,000 people are infected each year in Mexico, Central America and South America, accounting for at least 12,000 deaths per year [7]. It is estimated that there are approximately 326,000–347,000 Latin American-born infected individuals living in the U.S., however the number of autochthonous cases is unknown [8].

Reduviids, also known as triatomines or "kissing bugs", are blood-feeding insects that are the primary vector for CD transmission. Transmission generally occurs when an infected triatomine defecates near the bite site and the feces enters the wound, transmitting the parasite into the host. Triatomines may transmit the parasite to mammals, including humans [1], but can also infect reservoir hosts such as canines, opossums, raccoons, and other domestic [9, 10], and sylvatic animals [11]. Other transmission routes include oral ingestion of triatomine feces contaminated food or drink, congenital transmission from mother to a fetus, exposure to contaminated blood products and through organ transplantation from an infected donor [4]. Blood donation screening is the most common means by which individuals learn about their CD diagnosis in the U.S. [12].

CD includes two main phases: acute and chronic [1, 13]. Acute infections occur up to the first two months of the initial infection, which may manifest with mild flu-like symptoms or prolonged febrile illness [14, 15]. Other symptoms may include malaise; enlarged spleen, liver, and lymph nodes; localized or generalized edema; and chagomas or breaks in the skin [1]. Infection may also result in abnormal electrocardiogram (ECG) readings [10]. Acute infection may manifest as early as one-week post- exposure and may be self-limiting in most individuals [10]. The patient may not seek medical attention since the symptoms are mild and not unique to CD. During the chronic stage, two presentations are possible: the indeterminate form and determinate form. The indeterminate form is characterized as a chronic infection by *T. cruzi* without specific organ damage and is commonly asymptomatic. The determinant form is characterized as having specific organ damage with complications, which may include cardiac manifestation (e.g., cardiomyopathy, heart failure, altered heart rate or rhythm) and intestinal complications [13]. Approximately 70–80% of infected individuals [13, 16] will transition from the acute phase and remain in a latent or indeterminate chronic form of the disease (mostly asymptomatic), which may persist as a lifelong infection [4]. The danger of this asymptomatic status is that once symptoms do manifest, eliminating the parasite is either more difficult or impossible with the latter case resulting in death. Treatment of the disease is with anti-parasitic drugs (Benznidazole or Nifurtimox) [17]; however research does not conclusively suggest a reduction in mortality after treatment nor a reversal of symptoms if the patient has entered into the chronic phase with specific organ damage [18]. Approximately 20–30% of infected individuals will progress from the indeterminate chronic phase without organ damage to a “clinically evident disease” or chronic determinate phase. This progression occurs months to decades after becoming infected [15]. Infection in humans can present in many forms such as non-ischemic cardiomyopathy, heart failure, cardiac arrhythmias and sometime gastrointestinal disease [10]. Cardiovascular pathophysiology is believed to be multi-causal (e.g., direct parasitic aggression [19–22]). Sudden death due to cardiac complications can also occur [6]. For the scope of this study, heart-related symptoms were the primary focus.

Chagasic cardiomyopathy may include cardiac arrhythmias, heart failure, and risk of sudden death from ventricular fibrillation or tachycardia or thromboembolic events [10, 23]. Cardiovascular disease in CD patients is believed to be the result of the presence of the parasite in the cardiac tissue causing an immune-mediated myocardial injury [24]. A recent study by Hyson et al., screened 1156 patients for CD and revealed that out of the 23 patients that had positive serological screenings, cardiomyopathy and congestive heart failure was present in 43% of the cases [25]. CD may present as an idiopathic cardiomyopathy and therefore be overlooked by many or most healthcare providers as a diagnosis if they are inexperienced in seeing patients with CD. An estimated 10–15% of the total U.S. population (or 30,000 to 45,000 individuals) is estimated to be living with undiagnosed CD cardiomyopathy [26]. Many U.S. physicians and other healthcare providers (HCPs) are not well versed in CD screening, diagnostics, or treatment [27].

Between 2013 and 2019, 184 total cases of CD were reported in Texas [28]. Although regarded typically as a rare neglected tropical disease [1], current vector surveillance, [29, 30] the increased frequency of Chagas positive blood donors, [31–33] and population migration, [34, 35] demonstrate why more CD cases may be going undetected in the U.S.

With regards to U.S. physicians’ knowledge of CD, recent pre-post evaluation of healthcare providers (HCPs) in Texas suggests specific gaps in medical training and awareness on screening and diagnosing patients [36]. A separate study using a mixed methods approach supports these findings: HCPs were not confident overall in their skills to screen, diagnose, and treat CD patients [37]. In a recent study of sampled U.S. obstetricians, less than one-third of the study sample knew of the testing protocol, and one fifth knew of the follow-up protocols once a patient received a positive diagnosis [38]. Thus, facilitating CD education to healthcare providers remains a challenge [27, 36].

This study aims to illustrate potential heart-related CD in Texas using a geospatial and sensitivity analysis to inform health policy makers of the areas where prevention, education and screening efforts might be best served. Given the risk factors for exposure and transmission to CD and etiology of specific strains (i.e., heart-related symptoms), we hypothesize an increased number of suspected cases of CD throughout the state among younger Hispanic individuals. Based on the suspected case definitions (described below), we expect a higher proportion of individuals with undiagnosed CD with heart-related symptoms.

## Methods

### Data sources

The Inpatient Public Use Data File (IPUDF) for 2016, maintained by the Texas Department of State Health Services (TxDSHS), and the number of Chagas cases confirmed for 2019 by TxDSHS were used for geospatial analysis [39, 40]. In Texas, CD became a reportable condition in 2013; therefore hospital inpatient data from 2013 to 2016 were acquired. In Texas, a CDC confirmed diagnosis is reported to TxDSHS and is listed as either acute, chronic indeterminant or chronic determinant based on epidemiological investigations and interviews conducted by the local or regional health departments. Census data [41] was used to download the American Community Survey (ACS) 5-year Texas population estimates for 2016. This included Texas demographic data on age and Hispanic status by county. A base map was created by downloading the shapefile for the Texas counties from the U.S. Census Bureau (i.e., TIGER/Line Web interface) [42].

### Variables and case definitions

Patient demographics and patient diagnostic codes originated from the inpatient data (i.e., the raw quarterly base files from the PUDF, 2013 to 2016). The demographic variables included: patient’s age group (i.e., < 18; 18–44; 45–64; 65–74; and 75); ethnicity (Hispanic or non-Hispanic); race (American Indian/ Eskimo; Asian or Pacific Islander; Black; White; or Other); and sex code (male or female). Additional variables that were kept from the initial raw inpatient PUDF dataset included: record identification number for each hospital admission; patient’s county and zip code of residence; provider ID; and type of admission.

The admitting diagnosis and the twenty-four principle diagnostic codes were the queried variables from each quarterly base data file (e.g., IPUDF) for each hospital admission. The process was iterative and exploratory in identifying both the diagnosed CD cases and the potentially missed CD cases (undiagnosed) manifested through heart complications. These diagnoses variables were re-coded to determine if that particular patient record contained the ICD 9 or ICD 10 diagnostic codes of interest (e.g., Table 1: *CD cases*). A total of 3,088,978 hospital inpatient records were identified once all the datasets were combined (i.e., 2013–2016). A dichotomous variable (i.e., for any admission that had any of the CD-related ICD diagnosis codes) was created using the CD cases.

**Table 1:** Chagas disease and potential cardiomyopathy-related ICD 9 and ICD 10 codes with descriptions

Case	ICD Version	Diagnostic code	Description
Chagas disease	ICD-9-CM	086.0	Chagas with heart involvement
	ICD-9-CM	086.1	Chagas with other organ involvement
	ICD-9-CM	086.2	Chagas without mention of organ involvement
	ICD-10-CM	B57.0	Acute, heart
	ICD-10-CM	B57.1	Acute, without heart
	ICD-10-CM	B57.2	Chronic, with heart
Potentially missed CD cases (heart-related codes)	ICD-9-CM	414.8	Other forms of chronic ischemic heart disease
	ICD-9-CM	422.91	Idiopathic myocarditis
	ICD-9-CM	425.8	Cardiomyopathy, excludes Chagas
	ICD-9-CM	425.4	Cardiomyopathy, includes idiopathic
	ICD-10-CM	I25.5	Ischemic cardiomyopathy
	ICD-10-CM	I42.9	Cardiomyopathy, unspecified

A cardiologist with expertise in diagnosing CD was consulted with to identify and further review heart-related diagnostic codes (i.e., the potentially-missed CD cases). The list of ICD codes was then reviewed by a second cardiologist with experience in diagnosing and treating CD patients to eliminate unnecessary codes. The ICD diagnostic code and corresponding definition for heart-related cases, a proxy for potentially-missed CD diagnosis, are shown on the second half of Table 1. Additional ICD-10-CM codes for heart-related diagnoses were identified to expand the list of potentially-missed cases (Table 2). The IPUDF transitioned from utilizing ICD-9 diagnosis codes to ICD-10 in 2016. Additionally, this was the most recent year of available data for the analyses at the time of the study. The expanded list was only used to query 2016 to identify the potentially-missed CD cases. A dichotomous variable (i.e., for any admission that had any of the heart-related ICD diagnosis codes) was created using the heart-related ICD codes.

**Table 2:** Additional ICD-10 cardiomyopathy codes that could be potentially related to a missed Chagas disease diagnosis

Diagnostic code	Description
I428	Other cardiomyopathies
I429	Cardiomyopathy, unspecified
I4510	Unspecified Right Bundle Branch Block
I452	Bifascicular block
I441	Atrioventricular block, second degree
I442	Atrioventricular block, complete
I472	Ventricular tachycardia

### Data collection and management

The 2016 quarterly IPDUF base files were individually exported as a comma separated value file (CSV) to Excel. Exploratory data analysis and data cleaning were performed, and patient records that did not contain the case definitions were eliminated from the dataset. The combined raw IPUDF contained over 3 million hospital admissions of which 3.1% were included in this study, as they contained a heart related/CD diagnosis code. Demographic data for the state of Texas were downloaded using the American Fact Finder web application. The ACS estimates were chosen for the Hispanic population and age categories. Microsoft Excel was used for data cleaning. To calculate the Hispanic proportion, the number of Hispanics was divided by the total population for each county. To create the table for age groups, only the population estimates for males, females, and all, aged 20–59 were calculated.

### Mapping

Once definitions were finalized, the dichotomous variables for CD cases and potentially- missed CD (heart-related) were compiled, aggregated by year, and tabulated. ArcMap GIS (Version 10.6.0) was used to visualize the variables [43]. This software coupled with Excel 2016’s innate 3d mapping provided geographic descriptive capability. A series of maps were created to explore geographic areas of interest.

### Modeling

All but two of the 254 counties in the state were included in the models. Two of the 254 Texas counties were excluded in the models due to low reporting and patient county suppression, “The county code is suppressed if a county has fewer than five discharges for that quarter” [40]. Linear regression with residuals was applied using the following variables: male population, Hispanics, race (Asian or Pacific Islander; Black, White, and Other), county population, population density (persons per km^2^, and rate). The modelling techniques (e.g., Queen’s, Global Moran’s index, etc.) used can be referenced online: https://rpubs.com/R-Minator/chagas1. For instance, Moran’s index I captures (Li et al. 2007) the intercorrelation between two adjacent geographic units (counties or towns etc.) with respect to the CD incidence. The expected value of the Moran’s index is − 1/(N − 1) under the assumption of no geo spatial autocorrelation among the units. Where N is the number geographical units. An analysis on Global Moran’s I was used to account for location and variable differences [44]. Robust linear mixed-method models with Lagrange multiplier diagnostics as a way to test for spatial dependence [45]. A generalized spatial two-stage least squares model (using STSLS) was used. This model with weighted matrix accounted for variability. The model controlled for gender (males), age, and population.

## Results

### Descriptive statistics Chagas diagnoses and heart-related ICD codes

Utilizing the IPDUF database, 98 CD diagnoses between 2013 and 2016 were identified. Of these 98 cases, 78 presented with heart involvement and the other 20 were not specified. There were 366,575 cases that fit the ICD 9 or 10 definitions for potentially missed diagnosis (i.e., heart-related diagnoses). “Cardiomyopathy, including idiopathic” and “other chronic ischemic heart disease diagnoses” accounted for the most instances (118,206 and 150,207, respectively). The least occurring diagnostic code was for idiopathic cardiomyopathy (n = 384). Table 3 provides the descriptive statistics for the ICD-related data: total CD diagnoses and suspected (i.e., heart-related conditions).

**Table 3:** Descriptive statistics for Chagas disease cases and potentially missed Chagas disease cases using the ICD-related data from inpatient hospital records in Texas, 2013–2016

	Counties	Mean	SD	Median	Maximum	Total
CD cases	254	0.39	2.46	0	26	98
Potentially Missed CD cases (i.e., Heart-Related Conditions)	254	1443.21	4998.23	340.50	59,118	366,575

The data for the suspected cases (i.e., potentially missed CD cases) were zero-inflated. Of the 254 counties, only 15 were non-zero. The average number of CD cases in any county was 0.39, and the median was 0. The maximum number of confirmed CD inpatient cases was 26 (Dallas County). For total heart-related diagnoses (i.e., potentially missed CD), only 1 observation had zero cases (Loving County). The mean was 1443 cases with a median of 340.5 (large positive skewness). Within our sample, 49% were male and 39% were Hispanic. The largest Hispanic population resided in Starr County (99%). The smallest Hispanic population resided in Roberts County (3%). The median age was 34 years old.

### Maps

**Fig. 1:**
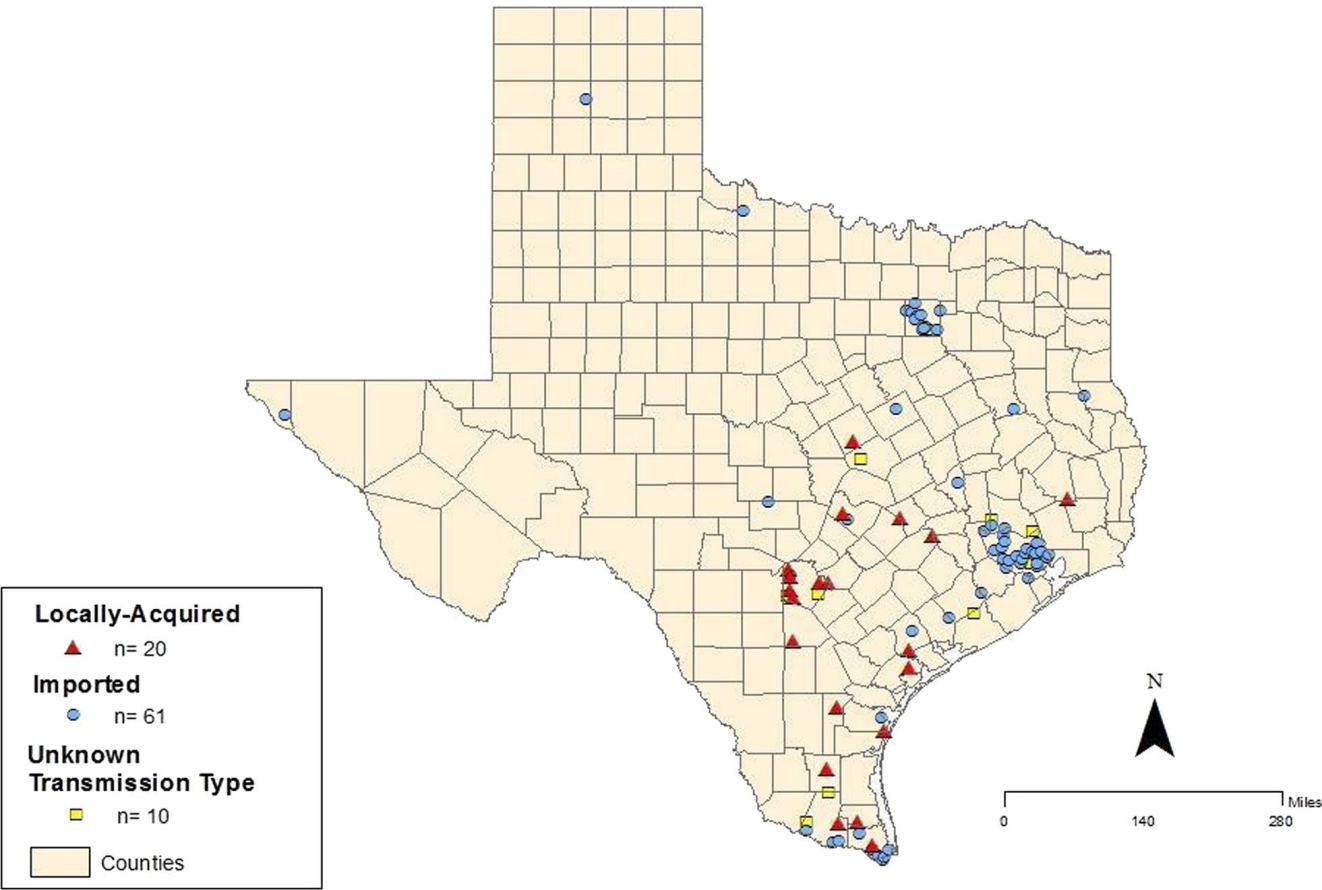
Chagas disease cases and transmission categories reported by the Texas Department of State Health Services by county of transmission from 2013 to 2016

Figure 1 illustrates the number of CD cases reported to TxDSHS between 2013 and 2016 in Texas, by transmission type as confirmed by the Zoonosis Control Division. This map provides a baseline for understanding geographic concentrations of confirmed cases of CD as reported by TxDSHS. A total of 91 individual cases were confirmed and reported, with each corresponding symbol representing a case. Based on the data presented in this figure, the largest clusters of imported cases were in Harris and Dallas counties. Individual imported cases were reported in Potter and Wilbarger counties in the north, El Paso in the far west, and Shelby and Anderson counties towards the east. Locally acquired cases were reported in Bexar County and some in South Texas counties of Hidalgo, Brooks, and Cameron. It should be noted that it is possible that locally-acquired cases could be congenital transmission. However, there is research and surveillance that indicates positive triatomines in South Texas have been frequently found near human dwellings, therefore autochthonous transmission is plausible [4, 46, 47].

**Fig. 2:**
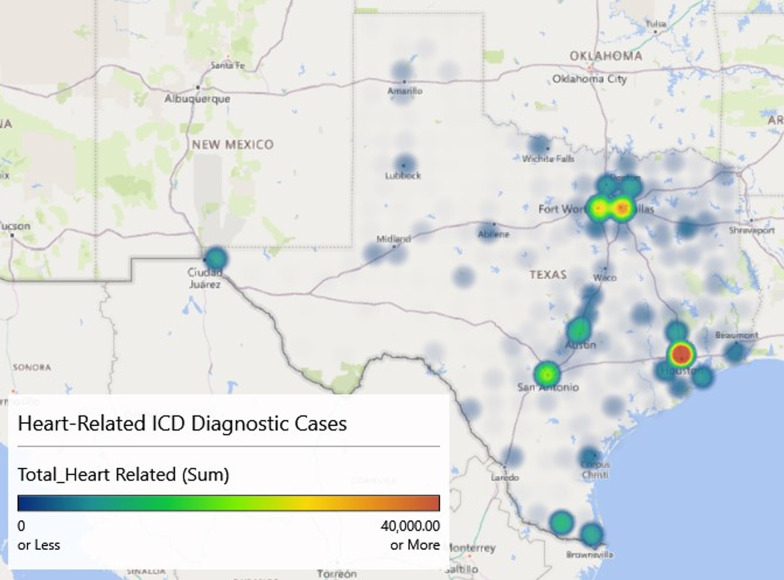
Heat map of ICD heart related codes that could be a possible missed Chagas disease diagnosis, 2013–2016

Figure 2 is a heat map of the ICD codes for heart-related diagnosis (i.e., potentially missed CD cases). The heat map provides color-changing values that indicate intensity levels of the disease processes (blue being the least intense and red being the most). From the map, it is clear that Houston has the most coded cases with Dallas, Fort Worth, and San Antonio ranking 2nd through 4th, respectively.

**Fig. 3:**
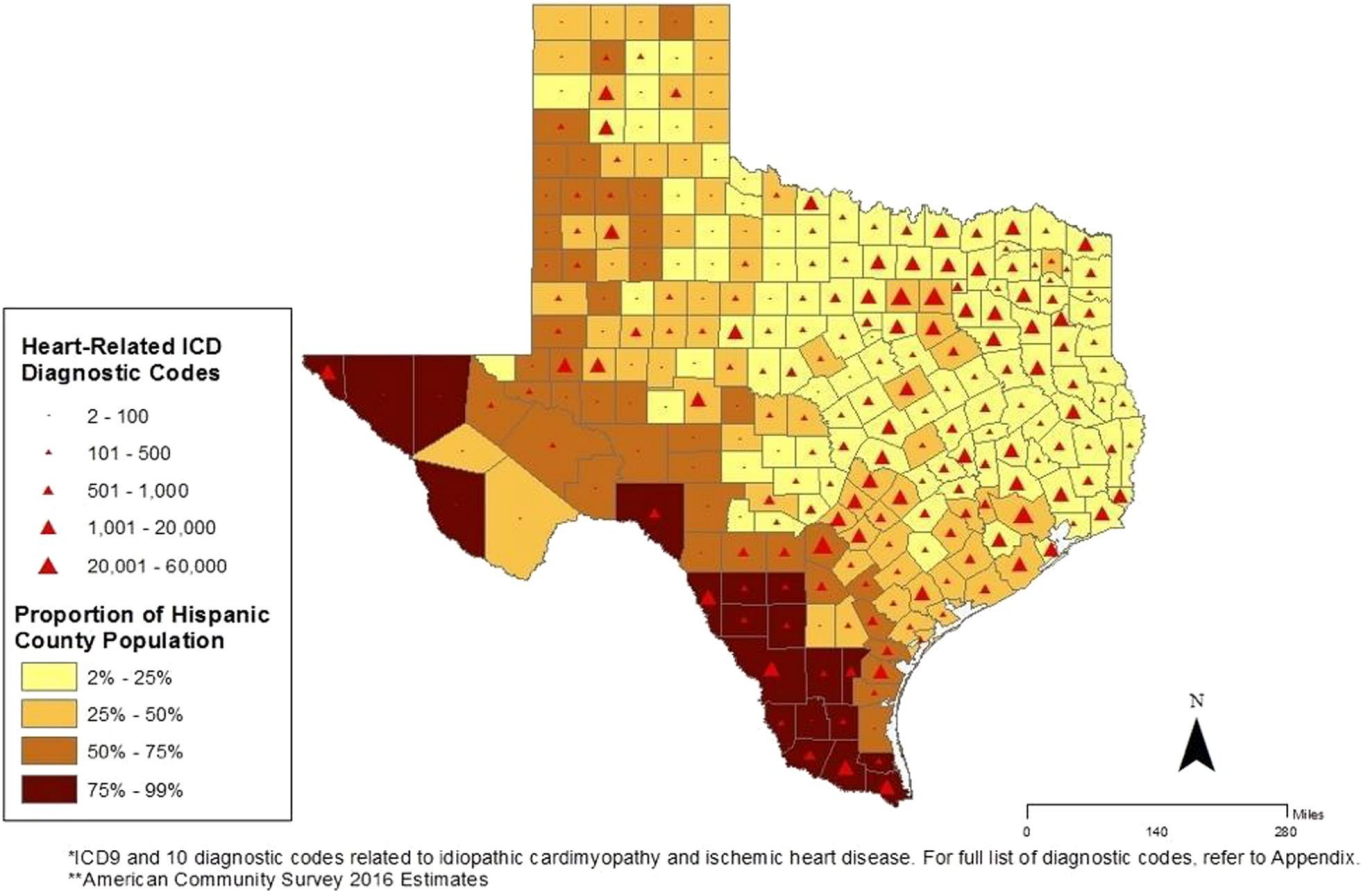
Potentially missed Chagas disease cases using heart-related ICD diagnosis codes from 2013 to 2016 and Hispanic population in Texas 2016

Figure 3 represents the ACS estimate of the Hispanic population. A graduated symbology (i.e., red triangles) is shown in both maps to illustrate the potential for missed CD disease diagnosis (displayed as the CD heart related diagnostic codes) given that a younger population might be experiencing heart complications. Counties with a high Hispanic population (75% to 99%) had a range of heart-related frequency, though the highest numbers were in El Paso, Maverick, Webb, Hidalgo, and Cameron. Counties with a lower proportion of a Hispanic population such as Randall or Montgomery had large (but not the highest) numbers of possible CD heart-related diagnostic codes. The increase in the triangle symbology in the population map can be seen in some counties with a large proportion of the population aged 20 to 59. In six counties with the largest proportion 55% to 65% in this age group (i.e., Hartley, Childress, King, Garza, Sterling, and Concho) had 100 or less heart-related diagnostic codes.

**Fig. 4:**
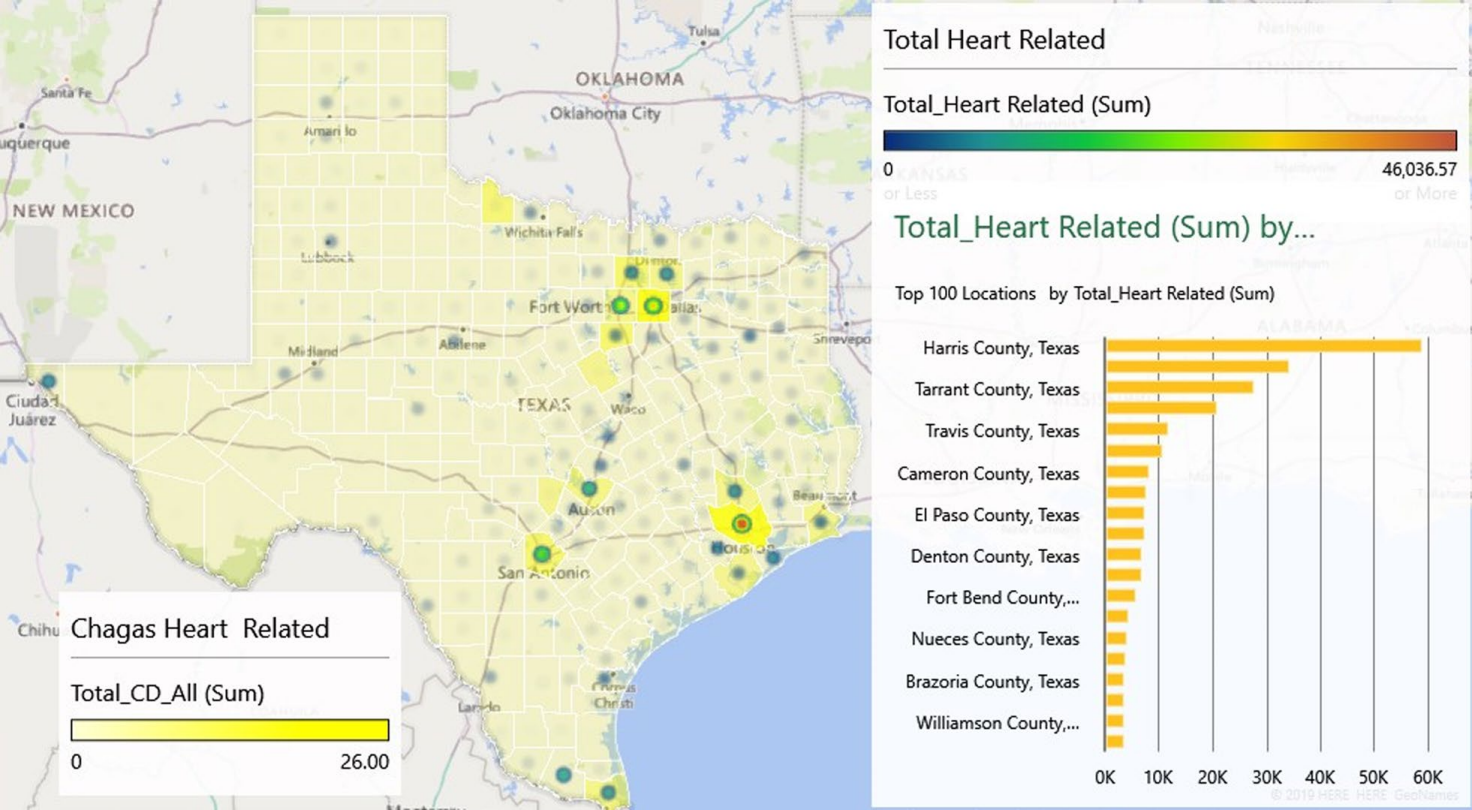
Heat map of Chagas disease diagnostic codes and heart-related ICD diagnostics codes combined for 2013–2016

Figure 4 shows the comparison between the CD diagnostic codes and the heat map for the number of heart-related codes. Harris and Dallas/Tarrant, Travis, Bexar, and Cameron counties show clusters of both ICD-coded CD and higher proportions of heart-related diagnostic codes. Wilbarger County near the Texas-Oklahoma border, a county with 28% Hispanic population, experienced six CD diagnoses yet only 345 heart-related codes.

### Statistical modeling

The mean number of total missed diagnoses was 347.6 with a SD of 1158. The descriptive statistics are presented in Table 4 for the additional variables. The first model (LM) resulted with an f statistic of 2.93, a coefficient determination of 0.07755 and a statistical significance (p-value < 0.05). An interactive map (https://rpubs.com/R-Minator/chagas1) was created using the model that shows the suspected rate (i.e., missed non-ICD Chagas diagnosis) per 100,000 of the county population.

**Table 4:** County-level descriptive statistics for linear model of possible missed diagnosis of Chagas disease in Texas

	Mean	SD	Median	Minimum	Maximum
Total	347.575	1157.378	76.500	1.000	12,983.000
Mean age	17.114	0.876	17.098	12.000	23.000
Male	165.278	540.208	39.500	0.000	6158.000
Female	123.310	408.708	25.500	0.000	4597.000
Hispanic	64.060	243.830	7.000	0.000	1944.000
Non-Hispanic	245.730	860.698	53.000	0.000	9888.000
Asian/Pacific Islander	1.274	6.847	0.000	0.000	80.000
Black	17.571	97.493	1.000	0.000	1217.000
White	69.944	194.634	16.000	0.000	1801.000
Other	13.202	60.067	1.000	0.000	787.000
Population	115,054.536	410,461.235	19,211.000	272.000	4,713,325.000
PopKM2	47.109	137.891	8.602	0.107	1166.873
Rate	394.257	159.947	385.607	35.868	1166.667

## Discussion

Interestingly, the findings in Fig. 4 are congruent with a previous study of Texas Blood donors, highlighting significant localities of concern [33].CD is more prevalent among individuals who have lived in endemic regions of Latin America and are considered to be at a higher risk for CD. However, it is estimated that only about 1% of the individuals living with CD in the U.S. are aware of their diagnosis [48]. In our study, heart disease symptoms that are common for patients with CD are used as a proxy to estimate possible cases and indicate areas to increase screening efforts. Areas in Texas with younger Hispanic populations and increased persons presenting with heart disease that could be related to CD was the focus of this paper [33]. It should be noted that this is not the only population at risk in Texas, however they are at a higher risk than other populations [48]. Presenting data in a visual and spatial format can often be useful in illustrating and contextualizing environmental factors [49]. Given that the CD vector is found throughout the State of Texas [50], local transmission is not well understood and the large Hispanic population of Texas, visualization of potential missed diagnoses is an important and significant exploratory analysis. In turn, through GIS analysis and data visualization, educational and outreach efforts can be further targeted throughout the state. The application of contextualizing maps and integrating with statistical methods to enhance public health activities has been described in chronic diseases [51]. However, the value and use of GIS to inform public health education activities on CD has not been previously studied.

The CD cases reported to TxDSHS are not homogenously dispersed throughout the state but occur in clusters and primarily in urban areas, where presumably there is increased access to physician care and larger populations. The policy implication is that screening for CD should begin with the populations most likely at risk [48, 52]. The data from TxDSHS [40] show the possibility for locally acquired or imported infection. Pockets of locally acquired cases were reported specifically in Bexar, Hidalgo, Brooks, and Cameron counties. However, no other areas, (i.e., the panhandle; western Texas including the El Paso region; and the eastern parts) show locally acquired infections. Moreover, five newly diagnosed CD patients are described in a case report [29]: All of the patients acquired CD locally and resided in rural Southeast Texas counties. This highlights the possibility of persons currently not knowing that they have CD because many cases remain undiagnosed, particularly since the disease can become latent. In addition to local transmission, Texas presents the opportunity to surveil and diagnose imported cases. It is imperative for HCP’s throughout the state to recognize CD and be able to screen and diagnose patients.

Some counties with a high burden of heart-related diagnosis are also areas with CD diagnosis. The congruence in the urban hubs (Bexar, Dallas, and Harris counties) reflects the overall population but may also reflect the availability and ability of physicians in those counties to recognize CD and appropriately screen, diagnose, and treat. Conversely the modeling provided new insight into geographic areas (i.e., Kenedy County that was not believed by the researchers to be an area if interest). More focused education and outreach could be targeted to healthcare providers who may have limited knowledge in screening and diagnosis of CD in geographic areas where we find that there could be a higher risk for CD along with noted elevations in heart disease among a higher population of Latinos. As of 2017, the estimated seroprevalence of CD in Mexico was 2.26%, much higher than previously thought [29]. A study in Starr County, Texas which lies adjacent to the Mexican border found that eight of 1196 study participants (0.7%) screened positive with 2 of the cases 1196 (0.2%) confirmed by study criteria [53]. With Texas and Mexico sharing such a large border region, it stands to reason that the seroprevalence in places such as Texas could be higher, however with limited surveillance and screening it is hard to know the true amount of CD in Texas or the U.S.

### Limitations

This research is one of a few examining CD through hospital records [53]. This is the first to examine statewide hospital records in order to qualify the potential for missed CD diagnosis in Texas. However, this research focused on potentially-missed diagnosed cases of chronic Chagas, rather than including acute and indeterminate chronic forms of CD. Moreover, in examining chronic CD, the scope of this research was limited to CCC, rather than looking at other sequelae (i.e., gastrointestinal complications). Furthermore, establishing the criteria for missed diagnoses of CD was the greatest challenge, given the lack of research to inform specific risk factors that account for CCC. Thus, the risk of misclassification is a concern.

Inpatient records were exclusively used, rather than including outpatient records given that CD patients do not necessarily require a hospitalization to be diagnosed. CD patients may be unaware of their CD status since the disease is asymptomatic. Because the patients are asymptomatic, they may be undiagnosed and thus not receiving appropriate care. Finally, the IPUDF data set does not provide unique patients, rather enumerates the records. Ultimately, this highlights the under-estimation of tur missed diagnoses of CD in Texas.

ICD-9 and ICD-10 heart-related and CD diagnostic codes were not completely comparable given differences in their definitions. While ICD-10 denotes the disease progression (i.e., acute, or chronic), there is no code specifying the indeterminate form of CD. In ICD-9 there is a code (086.2) that alludes to the asymptomatic, indeterminate form (i.e., Chagas without mention or organ involvement). Similarly, among the heart-related diagnostics, there is a cardiomyopathy, excluding Chagas in ICD-9 code but not one for ICD-10. Between 2013 and August of 2015, a total of 21 records indicated Chagas without mention of organ involvement. Furthermore, ICD codes are intended for medical billing and are not confirmed CD diagnosis. The identified barriers [55] to screening and diagnosing acute and chronic CD are documented further highlight the challenges in fully estimating the true prevalence of CD across the state. Our study utilized the hospital inpatient data to focus and target educational efforts throughout Texas.

### Recommendations

Future research can further explore the patterns of missed diagnoses within specific geographical targets. For example, examining and comparing urban and rural counties only in contrast to examining patterns throughout the state; or by examining differences in census tracts or zip codes). Furthermore, the case definitions for the missed CD diagnostic codes could be re-evaluated. For example, additional geospatial and statistical analyses can be performed on specific counties using only idiopathic cardiomyopathy diagnoses and comparing to other codes that accounted for the large number of potential CD heart-related diagnoses (e.g., other ischemic heart disease, ischemic cardiomyopathy, unspecified cardiomyopathy). Finally, additional research can map the county demographics and more specific risk factors for CCC (i.e., by narrowing the age group).

Secondly, the findings support the need for surveillance systems. An entomological surveillance system should include the study of natural infection in vectors. In turn, such systems could facilitate screening for individuals in communities with documented infestation. In the human population, a surveillance system would facilitate an increase in the accuracy, validity, and generalizability of a geospatial analysis. That is, maps that are created to illustrate the magnitude of CD cases in Texas would greatly benefit from epidemiological data that is specific to CD, rather than relying on administrative data such as the Texas PUDF. For example, recent findings have helped focus educational approaches, particularly using an Extension for Community Healthcare Outcomes (ECHO) model among community healthcare workers [56] in Texas.

## Data Availability

The modeling data that generated during the current study are available online: https://rpubs.com/R-Minator/chagas1*.* The coded ICD-9 and ICD-10 hospital data are not publicly available from the corresponding author on reasonable request. Source data (inpatient public use data file, through 2015) is available for direct download from the Texas Department of State Health Services: https://www.dshs.texas.gov/thcic/hospitals/Inpatientpudf.shtm. An official request must be submitted to request Year 2016.
